# Comparing the results of intradermal skin tests for four dust mite allergens in dogs with atopic dermatitis in Thailand

**DOI:** 10.14202/vetworld.2020.2381-2387

**Published:** 2020-11-09

**Authors:** Suttiwee Chermprapai, Pojnicha Chuayjuljit Anukkul, Teerawat Kritsadasima, Pudcharaporn Kromkhun, Naris Thengchaisri

**Affiliations:** 1 Department of Companion Animal Clinical Sciences, Faculty of Veterinary Medicine, Kasetsart University, Bangkok 10900, Thailand; 2 Dermatology Unit, Kasetsart University Veterinary Teaching Hospital, Bangkok 10900, Thailand; 3 Department of Physiology, Faculty of Veterinary Medicine, Kasetsart University, Bangkok 10900, Thailand

**Keywords:** allergy, atopic dermatitis, dogs, house dust mite, intradermal skin test

## Abstract

**Background and Aim:**

Hypersensitivity to house dust mites is a common cause of atopic dermatitis in dogs. The intradermal test (IDT) identifies allergens to be included in allergen-specific immunotherapy. Common mite allergens used for IDT include single source extracts obtained from *Dermatophagoides farinae* or *Dermatophagoides pteronyssinus* or multisource extracts from multimite species (mixed mites), as well as a combination of multimite species and proteins from feces and shed skin (house dust). The objectives of the present study were to evaluate the prevalence of mite sensitivity in dogs diagnosed with atopic dermatitis in different Thailand provinces and to determine if positive test results to mite allergens aligned.

**Materials and Methods:**

Eighty-two dogs (median age [range]: 5 years [11 months-11 years]; 51 males and 31 females) diagnosed with atopic dermatitis underwent IDTs with four different mite-related allergens (*D. farinae*, *D. pteronyssinus*, mixed mites, and house dust). The skin reactions were reported on a scale of 0-4 and the reactions 2+ were considered clinically relevant. The relationship between IDT results from different allergens was determined using Pearson’s correlation coefficient (r). Fisher’s exact test was used to compare IDT results for different mite allergens as well as for dogs residing in Bangkok versus other provinces in Thailand.

**Results:**

The prevalence (95% confidence interval [CI]) of positive IDT results for *D. farinae*, *D. pteronyssinus*, mixed mites, and house dust in dogs with atopic dermatitis was 64.63% (52.30-74.88), 58.54% (47.12-69.32), 47.56% (36.41-58.89), and 35.37% (25.12-46.70), respectively. A moderate correlation was found in IDT results between *D. pteronyssinus* and house dust (r=0.514), between *D. pteronyssinus* and *D. farinae* (r=0.426), and between *D. farinae* and mixed mites (r=0.423). The prevalence of dogs with positive IDT results for mite allergens with mono-sensitization, bi-sensitization, tri-sensitization, and quadru-sensitization did not differ significantly between dogs residing in Bangkok (11.63%, 18.60%, 25.58%, and 16.28%) and dogs residing in other provinces (12.82%, 30.77%, 35.90%, and 10.26%). The overall sensitivity (95% CI) and specificity (95% CI) of the mixed mites test associated with atopic dermatitis in dogs were 60.32% (47.20-72.40%) and 94.70% (74.00-99.90%), respectively. The overall sensitivity (95% CI) and specificity (95% CI) of the house dust test associated with atopic dermatitis in dogs were 42.90% (30.50-56.00%) and 89.50% (66.90-98.70%), respectively.

**Conclusion:**

House dust mites are an important source of allergens for dogs with atopic dermatitis. In the present study, no significant difference in the prevalence of atopic dermatitis was found in dogs living in the urban area compared with dogs living in the countryside. Application of multisource extracts from mites for IDT revealed a higher reaction to mixed mites than that of house dust.

## Introduction

Canine atopic dermatitis is a chronic multifactorial inflammatory and pruritic allergic skin disease associated with skin barrier dysfunction and imbalanced immune responsiveness against allergens [1,2]. Atopic dogs are commonly hypersensitive to house dust mites [1,3]. A diagnosis of canine atopic dermatitis can be made by taking the patient’s history, conducting a physical examination, and excluding other pruritic skin diseases; however, the intradermal skin test (IDT) remains a useful tool in specifying the causal allergen [4].

Allergen selection for IDTs varies depending on regional prevalence [4]. Common mite allergens used for IDTs include single source extracts obtained from *Dermatophagoides farinae* or *Dermatophagoides pteronyssinus* or multisource extracts from multimite species (mixed mites), as well as a combination of multimite species and proteins from feces and shed skin (house dust) [5,6]. The relationship between IDT results for various mite allergens remains unknown. Limited evidence is available to draw the conclusion that multisource extracts from mites potentially provide better or worse diagnostic value compared with single source extracts for IDTs. In addition, it has been suggested that allergy prevalence in humans in urban areas is higher than in the countryside [7]. Dogs living in the city in Thailand where pollution is higher than in rural areas may be related to higher positive IDT results for mite allergen. Positive IDT responses to multisource extracts from mixed mites should be differed from single mite allergen.

The objectives of the present study were to evaluate the prevalence of mite sensitivity in dogs diagnosed with atopic dermatitis in different Thailand provinces and to determine if positive test results to mite allergens aligned.

## Materials and Methods

### Ethical approval and informed consent

This study was carried out under the guidance of the Kasetsart University Institutional Animal Care and Use Committee (ACKU63-VET-044) and by the Ethical Review Board of the Office of National Research Council of Thailand (approval number U1-08491-2562). Informed owner consent was gained before commencing data collection on IDT testing.

### Animals, study location and period

Eighty-two client-owned dogs with atopic dermatitis (31 females and 51 males, median age of 4.3 years ranging from 11 months to 11 years) of different breeds, that visited the Dermatology Unit at Kasetsart University Veterinary Teaching Hospital (KUVTH) from 2015 to 2019, were included. The study was carried out with written consent from each dog’s owner. Of these 82 dogs, there were 37 small breeds (45.12%), 33 medium breeds (40.24%), and 12 large breeds (14.63%). In the small breed group, there were 11 Shi Tzu (29.73%), 7 Chihuahua (18.92), 5 Pug (13.51%), 4 crossbred (10.81%), 3 Poodle (8.11%), 2 Papillon (5.41%), 2 Dachshund (5.41%), 1 Jack Russel (2.70%), 1 Schnauzer (2.70%), and 1 Yorkshire Terrier (2.70%). In the medium breed group, there were 17 Beagle (51.52%), 4 crossbred (12.12%), 5 French Bulldog (5.15%), 2 Siberian (6.06%), 1 Shar Pei (3.03%), 1 Thai ridgeback (3.03%), 1 Thai Bangkaew (3.03%), 1 Weimaraner (3.03%), and 1 Welsh Corgi (3.03%). In the large breed group, there were 6 Labrador (50.00%), 5 Golden Retriever (41.67%), and 1 German Shepherd (8.33%). The dogs with atopic dermatitis met Favrot’s diagnostic criteria for atopic dermatitis, and other pruritic skin diseases were ruled out by direct examination of the ectoparasites, coat brushing, and skin scrapings [4]. Antiparasitic control was applied regularly. Skin cytology was performed to rule out inflammation caused by bacteria, yeast, and fungi. Adverse food reaction was ruled out by an elimination diet trial of 8-12 weeks. Intradermal skin test was performed to identify allergens for allergen-specific immunotherapy. No concurrent anti-inflammatory, antihistamine, antibacterial/fungal treatments, and essential fatty acids were allowed 4 weeks prior to the IDT.

### Intradermal skin testing

After the physical examination, each dog was sedated with dexmedetomidine (intramuscular, 40 μg/kg) and positioned in lateral recumbency. Intravenous fluid was given during the whole procedure. Prior to the test, the dog’s hair was clipped at one side of the lateral thorax (20×25 cm^2^) and the injection sites were marked at least 2 cm away from each other. The positive and negative controls and the allergenic extracts were each injected intradermally 0.05-0.1 mL with a 27-gauge needle.

Histamine phosphate (Histatrol®, ALK Abello, Port Washington, NY, 11050) diluted with buffered saline into 1:10,000 w/v was used as a positive control. The negative control was buffered saline that was also used to dilute the allergenic extracts. The concentrated extracts were purchased either from ALK Abello (Port Washington, NY, 11050) or Siriraj Medical Hospital, Faculty of Medicine, Mahidol University (Bangkok, Thailand). The diluted allergenic extracts were prepared according to the manufacturer’s recommendation or as previously described [5,6,8] as follows: *D. farinae* (100 PNU/mL), *D. pteronyssinus* (100 PNU/mL), mixed mites (100 PNU/mL prepared from *D. farinae* 50 PNU/mL plus *D. pteronyssinus* 50 PNU/mL), and house dust (1000 PNU/mL). The extracts were kept below 7°C and used within 2 months.

Skin reactions of erythematous wheals were evaluated on a score of 0 to 4+ based on the wheal size at 15 min after injection. Histamine phosphate (1:10,000 w/v), the positive control, was given a grade of 4+. Buffered saline, the negative control, was given a grade of 0. The diameters of skin reactions to the allergenic extracts were compared with the mean diameter and erythema of the wheal, developed in response to injection with the positive and negative controls [8], and subjective scores were recorded by dermatologists at the KUVTH. Reactions graded as 2+ were considered clinically relevant [9]. Numbers of positive skin reactions to four different allergens in each dog were evaluated on score of 0-4 using 0=non-sensitization, 1=mono-sensitization, 2=bi-sensitization, 3=tri-sensitization, and 4=quadru-sensitization, respectively.

### Statistical analysis

The data were analyzed using STATA12 (StataCorp, College Station, Texas, USA) and GraphPad Prism Version 6 (GraphPad Software, San Diego, California, USA). Sample sizes were calculated with a power of 80% and an alpha error of 5% to detect a difference between two proportions at 0.15. The prevalence of IDT results for different mite allergens as well as the prevalence of IDT results between dogs residing in Bangkok and other provinces in Thailand was compared using Fisher’s exact test. The relationship among IDT results for different allergens was determined using Pearson’s correlation coefficient (r). Receiver operating characteristic (ROC) analyses were used to compare the results for different mite-related allergens, and the sensitivity and the specificity of the test were evaluated. p<0.05 was considered statistically significant.

## Results

**Figure-1:**
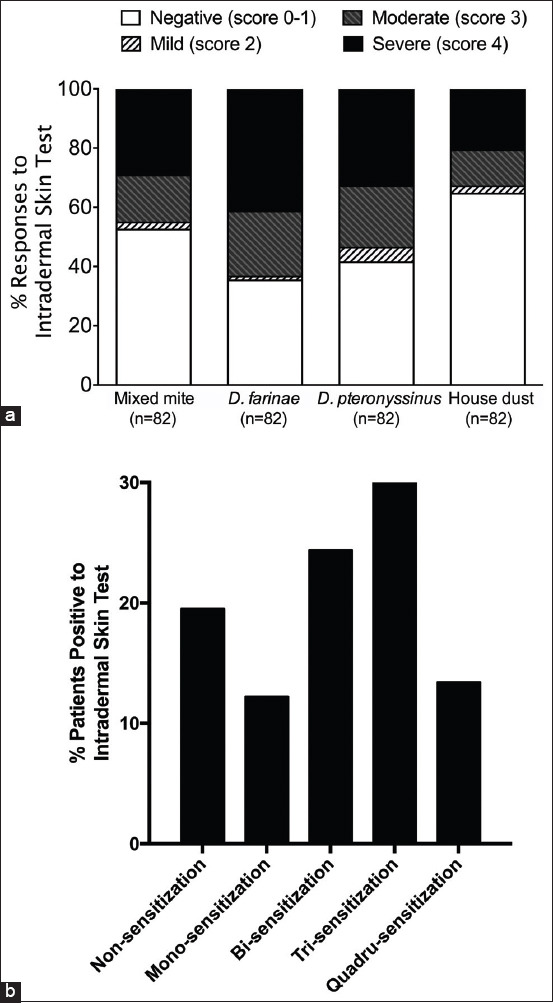
Severity of skin response in dogs to different allergens by intradermal skin test. (a) Responses to intradermal skin test and (b) percentage of patients positive to intradermal skin test.

The prevalence of positive IDT results for *D. farinae*, *D. pteronyssinus*, mixed mites, and house dust in dogs with atopic dermatitis was 64.63% (52.30-74.88), 58.54% (47.12-69.32), 47.56% (36.41-58.89), and 35.37% (25.12-46.70), respectively (Table-1). The prevalence of positive immune reactions to either *D. farinae* or *D. pteronyssinus* was 76.83% (66.20-85.44). The IDT results for the four different mite allergens are provided in Figure-1a. The prevalence of dogs with positive results for mite allergens classified as mono-sensitization, bi-sensitization, tri-sensitization, and quadru-sensitization was 12.20%, 24.39%, 30.49%, and 13.41%, respectively (Figure-1b).

**Table-1:** Summary of indoor allergen testing in dogs diagnosed with atopic dermatitis.

Allergen	Number of positive	Number of negative	% positive (95% CI)
*D. farinae*	53	29	64.63 (52.30, 74.88)
*D. pteronyssinus*	48	34	58.54 (47.12, 69.32)
House dust	29	53	35.37 (25.12, 46.70)
Mixed mites	39	43	47.56 (36.41, 58.89)
*D. farinae+D. pteronyssinus*	63	19	76.83 (66.20, 85.44)

CI=Confidence interval, *D. farinae=Dermatophagoides farinae, D. pteronyssinus=Dermatophagoides pteronyssinus*

**Figure-2:**
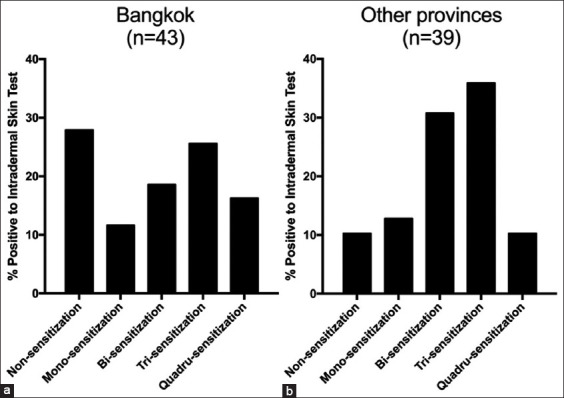
Percentage of dogs positive to intradermal skin test located in (a) Bangkok and (b) other provinces.

The influence of sex, breed, age, and coat length on allergic responses to different mite allergens was also evaluated. There was no significant difference in IDT results for the four mite allergens between males and females (Table-2). The IDT results were also not influenced by breed, age, and coat length (Table-3). In addition, there was no significant difference in IDT results for the four mite allergens in dogs residing in Bangkok compared with dogs residing in other provinces (Table-4). The prevalence of dogs with positive IDT results for mite allergens with mono-sensitization, bi-sensitization, tri-sensitization, and quadru-sensitization did not differ significantly between dogs residing in Bangkok (11.63%, 18.60%, 25.58%, and 16.28%) and dogs residing in other provinces (12.82%, 30.77%, 35.90%, and 10.26%; Figure-2).

**Table-2:** Influence of sex on specific indoor allergen testing in dogs diagnosed with atopic dermatitis.

Allergen	Male		Female		p-value
	
	Number of positive	Number of negative	Number of positive	Number of negative	
*D. farinae*	33	18	20	11	1.000
*D. pteronyssinus*	31	20	17	14	0.648
House dust	14	37	15	16	0.062
Mixed mites	25	26	14	17	0.821

D. farinae=Dermatophagoides farinae, D. pteronyssinus=Dermatophagoides pteronyssinus

**Table-3:** Effects of patient breed, age group, and coat length on allergen testing in dogs diagnosed with atopic dermatitis.

Category	Subtype	*D. farinae+D. pteronyssinus*		% positive	p-value

		Number of positive	Number of negative		
Breed	Small	29	8	78.38	
	Medium	25	8	75.76	1.000
	Large	9	3	75.00	1.000
Age group	Adult	49	17	74.24	
	Senior	14	2	87.50	0.338
Coat length	Short	43	13	76.79	
	Long	20	6	76.92	1.000

D. farinae=Dermatophagoides farinae, D. pteronyssinus=Dermatophagoides pteronyssinus

**Table-4:** Influence of geographic location on specific indoor allergen testing in dogs diagnosed with atopic dermatitis.

Allergen	Bangkok		Others		p-value
	
	Number of positive	Number of negative	Number of positive	Number of negative	
*D. farinae*	26	17	27	12	0.490
*D. pteronyssinus*	21	22	27	12	0.075
House dust	16	27	13	26	0.818
Mixed mites	19	24	20	19	0.658

D. farinae=Dermatophagoides farinae, D. pteronyssinus=Dermatophagoides pteronyssinus

A moderate correlation was found in IDT results positive for *D. pteronyssinus* and house dust (ρ=0.514), *D. pteronyssinus* and *D. farinae* (ρ=0.426), and *D. farinae* and mixed mites (ρ=0.423). A weak correlation was found in IDT results positive for *D. farinae* and house dust as well as for *D. pteronyssinus* and mixed mites (Table-5). Mixed mite allergens provided acceptable allergic responses to mites, with an area under the ROC curve of 0.7753 (Table-6). The overall sensitivity (95% confidence interval [CI]) and specificity (95% CI) of the mixed mites test associated with atopic dermatitis in dogs were 60.32% (47.20-72.40%) and 94.70% (74.00-99.90%), respectively. House dust provided acceptable allergic responses to mites, with an area under the ROC curve of 0.6617 (Table-6). The overall sensitivity (95% CI) and specificity (95% CI) of the house dust test associated with atopic dermatitis in dogs were 42.90% (30.50%-56.00%) and 89.50% (66.90%-98.70%), respectively.

**Table-5:** Pearson’s correlation coefficient (r) among intradermal skin test results in dogs diagnosed with atopic dermatitis.

	Mixed mites	*D. farinae*	*D. pteronyssinus*
House dust mite	0.086 (–0.119, 0.346)	0.233* (0.064, 0.489)	0.514** (0.364, 0.718)
Mixed mites	–	0.423** (0.237, 0.618)	0.373** (0.210, 0.587)
*D. farinae*	–	–	0.426** (0.288, 0.676)

* p<0.05,

** p<0.01, – indicate self correlation or duplicate results

**Table-6:** Area under the ROC curve of multisource mite allergens for IDT.

Allergen	AUC (fit model)	Sensitivity, % (95% CI)	Specificity, % (95% CI)
Mixed mites	0.7753	60.32 (47.20-72.40)	94.70 (74.00-99.90)
House dust	0.6617	42.90 (30.50-56.00)	89.50 (66.90-98.70)

AUC=Area under curve, CI=Confidence interval, IDT=Intradermal skin test, ROC=Receiver operating characteristic

## Discussion

In the present study, the IDT was performed in dogs with atopic dermatitis using different mite allergens. Positive IDT responses were found to be highest for *D. farinae* (64.63%), followed by *D. pteronyssinus* (58.54%), mixed mites (47.56%), and house dust (35.37%). There was a low to moderate correlation between the results of IDTs using a single mite allergen (*D. farinae* or *D. pteronyssinus*) and multimite species (mixed mites or house dust). Nonetheless, the results of IDTs for the two different multimite allergens were not correlated (q=0.086, p>0.05). There was also no difference in the allergic responses of dogs with atopic dermatitis to different mite allergens based on sex, breed, age, and coat length. In addition, there was no significant difference in IDT results for the four mite allergens in dogs residing in Bangkok compared with dogs residing in other provinces. The prevalence of dogs with positive IDT results for mite allergens with mono-sensitization, bi-sensitization, tri-sensitization, and quadru-sensitization was also not significantly different between dogs residing in Bangkok and dogs residing in other provinces. IDT provides positive reactions to mixed mites allergens but not as strong as individual allergen. The single allergen should be better included in the IDT and allergen-specific immunotherapy. The present findings also suggest that dogs with positive IDT to mite allergens are possibly hypersensitive to more than 1 allergen.

Diagnosing canine atopic dermatitis involves interpreting clinical features and ruling out other allergic skin diseases. It is recommended to confirm and identify the relevant allergens by the IDT or allergen-specific immunoglobulin E (IgE) serology tests [4]. The IDT gives an immediate cutaneous response and relates to cutaneous mast cell reaction, whereas the circulating allergen-specific IgE measurement may not be related to the clinical signs or the results of IDT [10-12]. The best advantage of IDT is that its results respond directly to the allergen penetrating the skin [8]. The most common allergens causing canine atopic dermatitis in several countries are house dust and storage mites, epidermal extracts, insects, fleas, molds, and pollens, and their prevalence differs between geographical locations including the United States, Europe, and Asia [4,9]. One study reported in 2008 that the most common allergens in Thailand are *D. farinae* and *D. pteronyssinus* [5].

Bangkok is the capital of Thailand and is located in the center of the country. The city has grown rapidly and becomes a hub for the economy, business, politics, and education. Thus, many Thai people from other provinces have relocated to Bangkok, leading to severe traffic congestion and air pollution in the city [13]. Studies focused on humans have described that living in urban areas where pollution is higher than in rural areas may be related to a higher prevalence of allergies [7]. It has been suggested that the environment also plays a role in atopic dermatitis development and allergic responses in dogs [3]. In the present study, no significant differences in the prevalence of atopic dermatitis were found in dogs living in Bangkok compared with dogs living in other provinces. This finding contrasts earlier and more recent studies indicating that dogs living in rural areas are at lower risk of developing atopic dermatitis [14-16]. This discrepancy may be explained by the small sample size and/or the concentration of allergens evaluated in this study.

A few decades ago, the effects of age, breed, and sex on the prevalence of canine atopic dermatitis were under debate. The prevalence of canine atopic dermatitis varies between 3% and 15% depending on breed, geographic location, and exploratory method [17-21]. Recently, one study that included two closely related dog breeds revealed that the hypersensitivity to similar allergens was influenced by sex, neutering status, and differing coat color within the same breed [14]. Breed predisposition may vary according to geographic location and overrepresentation of the studied areas [17,18]. In the current study, no significant differences in the IDT results for the mite allergens were observed based on sex, breed, age, or coat length. This may be due to the small number of patients and crossbreed dogs that were enrolled in the current study; thus, the effects of these factors on IDT results should be evaluated further in a larger study.

The allergenic extracts used for the mixed mites in this study were prepared at half of the concentration compared with that of the individual allergens, which may have decreased the reactivity of the IDT, hence lowering the sensitivity of the immune reactions. This finding is in agreement with an earlier study that also reported higher false-negative results for tests with mixed allergens [22]. In addition, the positive immune reactions to house dust allergens in this study were lower than the positive reactions detected to the individual house dust mite. The variable positive reactions might be due to the combination of several protein sources such as shredded skin, house dust mites, insect debris, and bacteria in the house dust mixture that may not provide as strong antigenicity compared with the individual allergen tested separately [8]. The differences in positive immune reactions in IDTs can depend on the type and source, the concentration, and the preparation of the allergenic extracts [8]. The differences can also be influenced by the individual variation in the host’s immune response.

The limitations of this study include the lack of IDT results for storage mite allergenic extract. Positive reactions to house dust mites possibly caused by cross-reactivity of storage mites, as previously described, cannot be ruled out [23]. Another limitation of this study was the concentration of *D. pteronyssinus* allergenic extract, which was prepared at the lower limit of an optimal concentration that was later revised [6]. This may have caused false-negative reactions of IDT to *D. pteronyssinus* and consequently may have affected the findings on atopic dermatitis development influenced by living in or outside of Bangkok. In people, bedding has been suggested to be one of the most important sources of mite allergens. Different mattress materials have been shown to influence the accumulation rate of mite allergens [24]. The role of pet bedding in atopic dogs remains elusive and selecting the appropriate materials for dog bedding may play a pivotal role in management of atopic dermatitis in dogs allergic to mite allergens.

## Conclusion

House dust mites are an important source of allergens for dogs with atopic dermatitis. The application of multisource extracts from mixed mites for IDT in dogs with atopic dermatitis did not provide cutaneous allergenic responses as strong as of individual mite extracts. The prevalence of atopic dermatitis among dogs residing in urban versus rural areas in Thailand should be further investigated. Several factors, internal and external to a patient can affect the sensitivity of the IDT.

## Authors’ Contributions

SC developed the concept, designed the study, performed the experiment, performed statistical analysis, and wrote the manuscript. PCA collected the IDT samples. TK collected the IDT samples. PK collected the IDT samples. NT designed the study and performed statistical analysis. All authors contributed to the drafting and revision of the manuscript. All authors read and approved the final manuscript.
